# Sensitivity of nasal airflow variables computed via computational fluid dynamics to the computed tomography segmentation threshold

**DOI:** 10.1371/journal.pone.0207178

**Published:** 2018-11-16

**Authors:** Giancarlo B. Cherobin, Richard L. Voegels, Eloisa M. M. S. Gebrim, Guilherme J. M. Garcia

**Affiliations:** 1 Department of Ophtalmology and Otorhinolaryngology, Universidade de São Paulo, São Paulo, Brazil; 2 Department of Radiology, Hospital das Clínicas da Faculdade de Medicina da Universidade de São Paulo, São Paulo, Brazil; 3 Department of Biomedical Engineering, Marquette University & The Medical College of Wisconsin, Milwaukee, Wisconsin, United States of America; 4 Department of Otolaryngology and Communication Sciences, Medical College of Wisconsin, Milwaukee, Wisconsin, United States of America; Worcester Polytechnic Institute, UNITED STATES

## Abstract

Computational fluid dynamics (CFD) allows quantitative assessment of transport phenomena in the human nasal cavity, including heat exchange, moisture transport, odorant uptake in the olfactory cleft, and regional delivery of pharmaceutical aerosols. The first step when applying CFD to investigate nasal airflow is to create a 3-dimensional reconstruction of the nasal anatomy from computed tomography (CT) scans or magnetic resonance images (MRI). However, a method to identify the exact location of the air-tissue boundary from CT scans or MRI is currently lacking. This introduces some uncertainty in the nasal cavity geometry. The radiodensity threshold for segmentation of the nasal airways has received little attention in the CFD literature. The goal of this study is to quantify how uncertainty in the segmentation threshold impacts CFD simulations of transport phenomena in the human nasal cavity. Three patients with nasal airway obstruction were included in the analysis. Pre-surgery CT scans were obtained after mucosal decongestion with oxymetazoline. For each patient, the nasal anatomy was reconstructed using three different thresholds in Hounsfield units (-800HU, -550HU, and -300HU). Our results demonstrate that some CFD variables (pressure drop, flowrate, airflow resistance) and anatomic variables (airspace cross-sectional area and volume) are strongly dependent on the segmentation threshold, while other CFD variables (intranasal flow distribution, surface area) are less sensitive to the segmentation threshold. These findings suggest that identification of an optimal threshold for segmentation of the nasal airway from CT scans will be important for good agreement between *in vivo* measurements and patient-specific CFD simulations of transport phenomena in the nasal cavity, particularly for processes sensitive to the transnasal pressure drop. We recommend that future CFD studies should always report the segmentation threshold used to reconstruct the nasal anatomy.

## Introduction

Computational fluid dynamics (CFD) technology has great potential as an objective tool in rhinology, given that it can quantify all the main physiological functions of the nose, including airflow conductance, delivery of odorant molecules to the olfactory cleft, and heating, humidification, and filtration of inspired air [1–5]. CFD studies have demonstrated that these physiological functions are impaired in several nasal pathologies. For example, nasal airway obstruction (NAO) is associated with a reduction in airflow conductance and inspiratory mucosal cooling [6, 7], while nasal dryness in empty nose syndrome is associated with a reduction in the surface area available for moisture exchange [8]. One of the most attractive features of CFD technology is the ability to perform virtual surgery and thus to predict the optimal surgical intervention for each patient [9]. For example, Hariri and colleagues [10] applied virtual surgery to reduce gradually the size of the inferior turbinate in 5 patients with NAO. The authors found that inferior turbinate reduction (ITR) reduced nasal resistance in 3 patients who had a high resistance in the turbinate region pre-operatively, but ITR had a minimal effect on nasal resistance in 2 patients. These studies illustrate how CFD-based virtual surgery planning has the potential to improve the outcomes of nasal surgery [9–12].

One critical step that has received little attention in the CFD literature is the segmentation of the nasal cavity and paranasal sinuses from medical images [13]. Segmentation is the process by which 3-dimensional (3D) models of the airspace are created from magnetic resonance images (MRI) or computed tomography (CT) scans [14]. Segmentation is critical for CFD analysis of nasal airflow because the nasal cavity is narrow (typically 2–3 mm wide), thus minor differences in nasal geometry can lead to substantial differences in airflow variables. For example, assuming that a CT scan with 0.4 mm pixel size was obtained from a nasal cavity that is 3 mm wide, an imprecision of 1 pixel on each side of the airway corresponds to an error as large as 27% for the nasal cavity width. CT scans have a higher contrast between air and soft tissues than MRI, but the precise location of the air-tissue boundary is still limited by the CT resolution and often can be unclear. Most medical imaging software use a constant threshold in Hounsfield Units (HU) to segment the nasal airspace. Conventionally, air has a CT number of -1000HU, while water has a CT number of 0HU [15]. Since a minimum amount of noise is inherent to every CT image [16], air is better represented by a CT number interval rather than a single value [17]. For segmentation of the upper airway, the lower limit of this range is usually set at -1000HU or a similar number depending on the software specifications [18]. On the other hand, the upper limit of the threshold range is still open to debate [14, 18–20]. In fact, the optimal threshold for airway segmentation may vary for different CT scanners, CT protocols, airway regions, and patient-specific anatomy [15, 18]. Surprisingly, the threshold for segmentation of the nasal airspace is rarely reported in the nasal CFD literature.

The objective of this study is to estimate the imprecision in CFD-derived nasal airflow variables caused by inaccurate segmentation of the nasal airway from CT scans. Three-dimensional models of the nasal airspace were created for three NAO patients after mucosal decongestion. For each patient, nasal airway reconstructions were created using three different values of the CT number threshold (-800HU, -550HU and, -300HU). Our results demonstrate that some CFD variables are strongly dependent on the threshold used for airway segmentation, while other CFD variables are less sensitive to the segmentation threshold. These findings suggest that identification of an optimal threshold for segmentation of the nasal airway from CT scans will be important for quantitative agreement between *in vivo* measurements and CFD simulations of transport phenomena in the nasal cavity, particularly for phenomena governed by the pressure drop across the nasal cavity.

## Methods

### Patient cohort

This study was approved by the IRB committee of the Hospital das Clínicas of Universidade de São Paulo, Brazil (approval number: 0738/11). Informed consent (both written and verbal) was obtained from each patient. Three patients with nasal airway obstruction (NAO) due to structural abnormalities were studied. Each exam represents a distinct CT machine and protocol used routinely in our Radiology Department (Table 1). All patients had failed clinical management and were scheduled to undergo NAO surgery at the Department of Ophtalmology and Otorhinolaryngology of the Universidade de São Paulo. Pre-surgery CT scan was indicated to investigate symptoms not elucidated by routine clinical exams, such as headache and post nasal drip sensation.

**Table 1:** Patient demographics and CT information.

Patient	#1	#2	#3
**Age/Gender**	59Y/F	36Y/M	49Y/M
**Scanner**	Toshiba/Aquilion	GE/Discovery	GE/Lightspeed
**Algorithm**	FC30	Bone	Bone
**Pixel size (mm)**	0.287	0.346	0.332
**Slice thickness (mm)**	0.5	0.625	1.250
**Slice increment (mm)**	0.3	0.625	0.6

Different CT scanners and protocols were used for CT acquisition in each patient.

### Rhinomanometry

*In vivo* measurements of nasal resistance were performed with rhinomanometry following the Standardization Committee recommendations [21]. All participants waited for at least 20 minutes in a resting room prior to application of a topical vasoconstrictor drug (oxymetazoline 0.05%, two sprays of 0.1ml in each nostril followed by an extra spray 5 minutes later). Anterior active rhinomanometry (Rhinomanometer NR6, GM Instruments, Kilwinning, UK) was performed 15 minutes after the second oxymetazoline spray. CT scanning was performed approximately 90 minutes after rhinomanometry.

### Creation of anatomic models

Semi-automatic segmentation of the nasal airspace was performed in Mimics 16.1 (Materialise Inc, Leuven, Belgium). Reconstructions of the nasal airspace were created with three different thresholding ranges, namely -1,024 to -800 HU, -1,024 to -550 HU, and -1,024 to -300 HU. In other words, the lower limit of -1,024 HU (lowest CT number in Mimics) was used for all models, while the upper limit was varied (-800, -550, and -300 HU). This allowed us to investigate systematically how the airspace segmentation affects the CFD variables. The 3D reconstructions included the nasal airspace from nostrils to nasopharynx, excluding all paranasal sinuses to reduce computational costs.

The 3D models were exported to ICEM-CFD 14.0 (ANSYS Inc., Canonsburg, PA) where a spherical surface intersecting the face was defined as the inlet. The outlet was a planar surface created at the pharynx. A mesh with approximately 4 million tetrahedral elements was generated for each model. This mesh size was defined based on a mesh independence test. Mesh quality was ensured by having all tetrahedral cells with aspect ratio greater than 0.3 to avoid distorted elements.

### Computational fluid dynamics simulations

Steady-state inspiratory airflow simulations were conducted in Fluent 14.0 (ANSYS Inc). Airflow was assumed to be laminar based on experimental observations that nasal airflow is mostly laminar for bilateral flowrates less than 20 L/min [22–24]. The following boundary conditions were applied: (1) air velocity set to zero at all walls, (2) inlet pressure set to atmospheric pressure (i.e., zero gauge pressure), and (3) outlet gauge pressure set to -30, -20, or -10Pa. Numerical convergence was defined as stability of the flowrate monitored at the outlet and residuals of continuity and velocity equations falling below 0.001. Separate simulations were performed of airflow in the left cavity and right cavity, respectively, to replicate the rhinomanometry protocol for measuring unilateral nasal resistance. One nostril was assumed to be occluded (wall boundary condition), while simulating inspiration through the contralateral nostril. These simulations reproduce the anterior rhinomanometry protocol where one nostril is occluded with a tape and the pressure probe is pierced through the tape to measure the transnasal pressure drop.

Unilateral nasal resistance (R = ΔP/Q) was calculated as the ratio of the pressure drop (ΔP) from inlet to nasal choana to the unilateral airflow rate (Q). In rhinomanometry, nasal resistance is typically measured at a transnasal pressure drop of 75 Pa or 150 Pa [21]. However, in this study, nasal resistance was computed at a unilateral flowrate of 125 ml/s, a flowrate typically used in CFD studies to simulate breathing at rest [25]. To compute nasal resistance at a unilateral flowrate of 125ml/s, a power law curve (ΔP = a*Q^b^, where a and b are constants) was used to fit the CFD results and to estimate the transnasal pressure drop associated with a unilateral flowrate of 125ml/s.

Cross-sectional areas of coronal sections perpendicular to the nasal floor were also calculated in ANSYS Fluent. The relative distance from nostrils (D) was defined as D = (z-z_nostril_)/L_septum_, where z is the position of a coronal section, z_nostril_ is the position of the first coronal section after the nostrils, and L_septum_ is the length of septum. Numerical results were exported to FieldView 15.1 (Intelligent Light Inc., Rutherford, New Jersey), where streamlines and flow allocation were analyzed.

## Results

### Segmentation of the nasal cavity from CT scans

**Fig 1:**
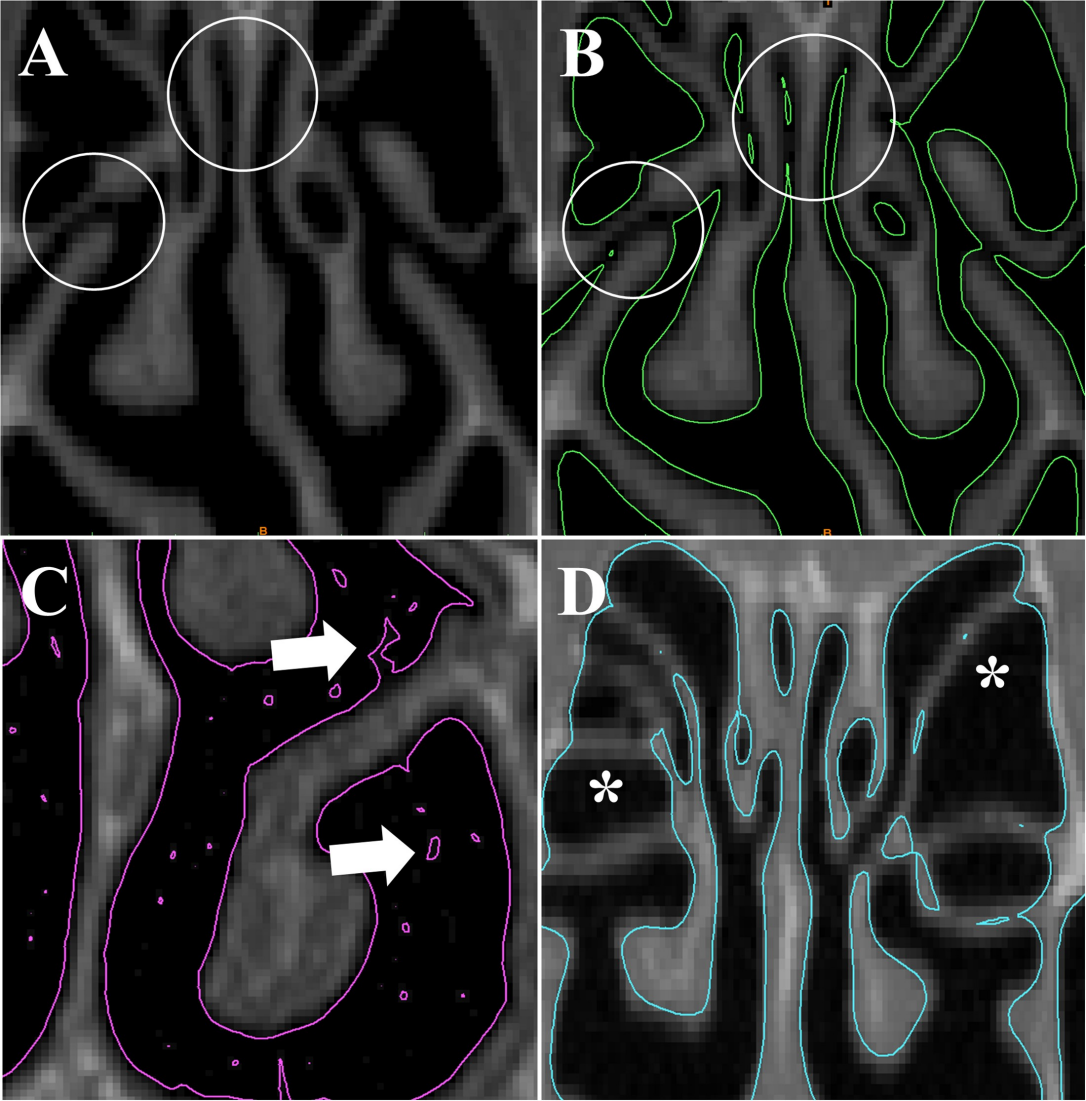
Common artifacts observed when segmenting the nasal cavity from CT scans. (A) Coronal CT of patient #2 shows a patent ostiomeatal complex and a patent olfactory cleft (circles). (B) Contours of 3D reconstruction created with thresholding range -1024HU to -950HU show discontinuities in the airspace (circles). (C) In patient #1, the 3D reconstruction created with the same thresholding range has surface irregularities and noise (arrows). (D) In patient #3, the 3D model created using the thresholding range -1024HU to -200HU did not segment the walls of the ethmoid sinuses correctly.

An exploratory investigation was first performed to identify which thresholding ranges provide acceptable reconstructions of the nasal airspace. In our sample of 3 NAO patients after mucosa decongestion, when a very narrow interval around the theoretical CT number of air (-1000HU) was used, namely -1024HU to -950HU, narrow regions of the nasal cavity of patient #2 were not segmented correctly, leading to many disconnected regions in the 3D reconstruction (Fig 1A and 1B). In patient #1, this thresholding range provided a 3D reconstruction with many blobs of “floating tissue" inside the airspace due to noise in the CT numbers (Fig 1C). Although these “floating tissue" artifacts could be easily corrected using the function "fill holes" in Mimics, noise near the walls created surface irregularities that could not be corrected with semi-automated tools, which would require a significant amount of hand-editing to correct (Fig 1C). Comparison between Fig 1B and 1C demonstrates how the same CT number interval can result in different 3D reconstruction shortcomings depending on the CT quality and the anatomic region where the threshold is applied. At the other extreme of the thresholding range, an upper limit of -200HU incorrectly identified thin walls as air, leading to unrealistic segmentation in the ethmoid sinuses of patient #3 (Fig 1D).

**Fig 2:**
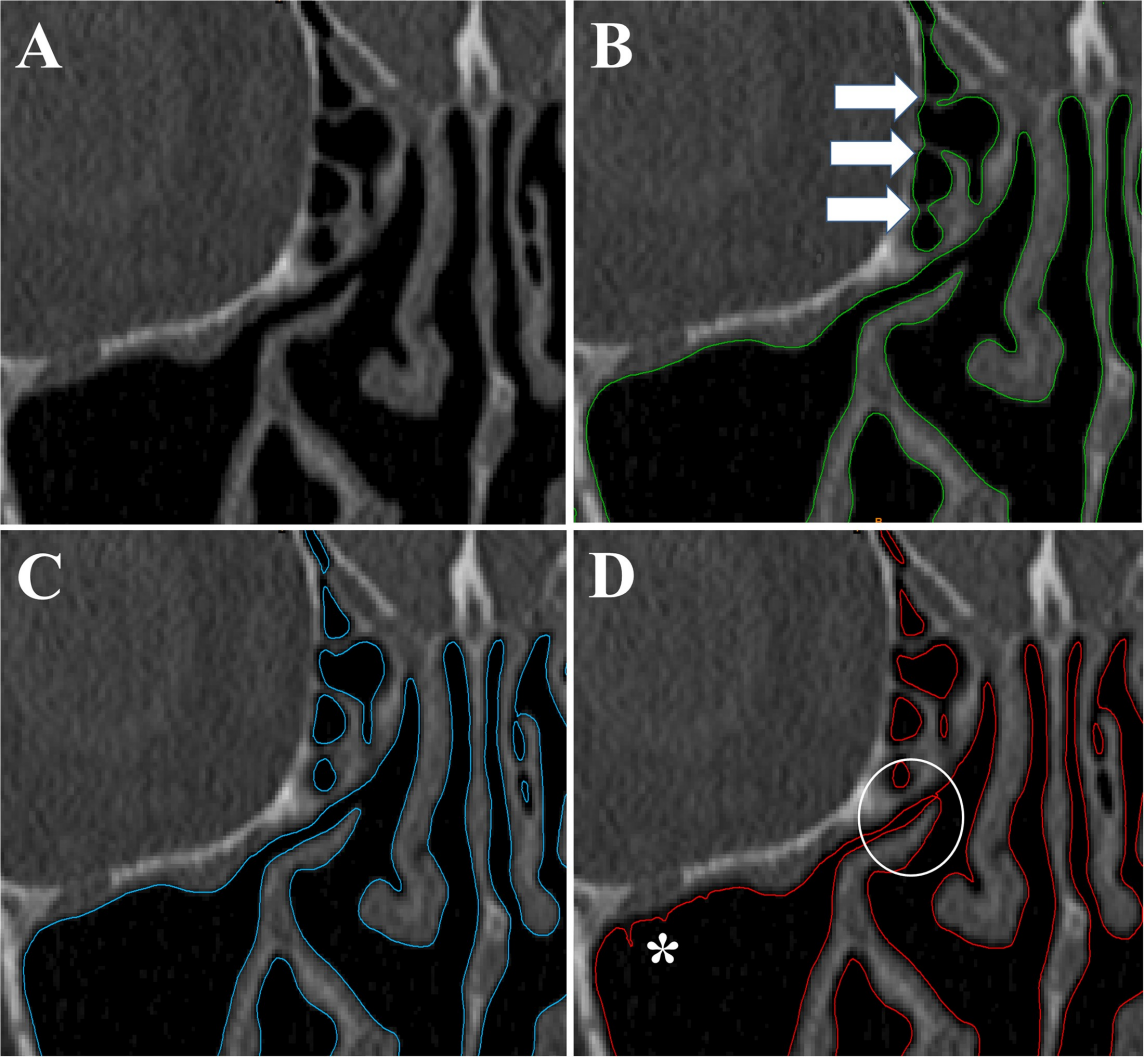
Effects of segmentation threshold on 3D reconstruction of the nasal cavity. A wide range of segmentation thresholds (namely -800HU to -300HU) provides acceptable 3D reconstructions of the human nasal cavity. (A) Coronal CT of patient #1 after mucosal decongestion showing the middle turbinate, ethmoid sinuses, and part of the right maxillary sinus. (B,C,D) Contours of 3D models created using three different thresholds (-300 HU, -550 HU and, -800 HU). At the upper limit of acceptable thresholds (-300 HU, panel B), thin soft tissue walls are incorrectly identified as air (arrows). At the center of the range (-550 HU, panel C), a good 3D reconstruction is obtained with few or no artifacts. At the lower limit of acceptable thresholds (-800HU, panel D), narrow passages become partially or completely obstructed (circle) and irregularities appears at air-tissue boundary (asterisk).

Based on this preliminary exploration, we concluded that acceptable reconstructions of the nasal airspace could be obtained without excessive amounts of hand-editing using values between -800HU and -300HU for all three CT scans. The maximum value (-300HU) still sometimes failed to segment thin walls (Fig 2A and 2B), while the minimum value (-800 HU) sometimes failed to capture narrow passages (Fig 2D). Thus, these extremes values (-300HU and -800HU) still required some hand-editing to obtain a smooth 3D reconstruction, but were selected to represent the maximum possible uncertainty in the segmentation threshold. When a value of -550HU was used, the number of segmentation artifacts was minimal and thus smooth 3D reconstructions could be obtained with little hand-editing (Fig 2C).

### Effect of segmentation threshold on cross-sectional areas, surface area, and volume of the nasal cavity

**Fig 3:**
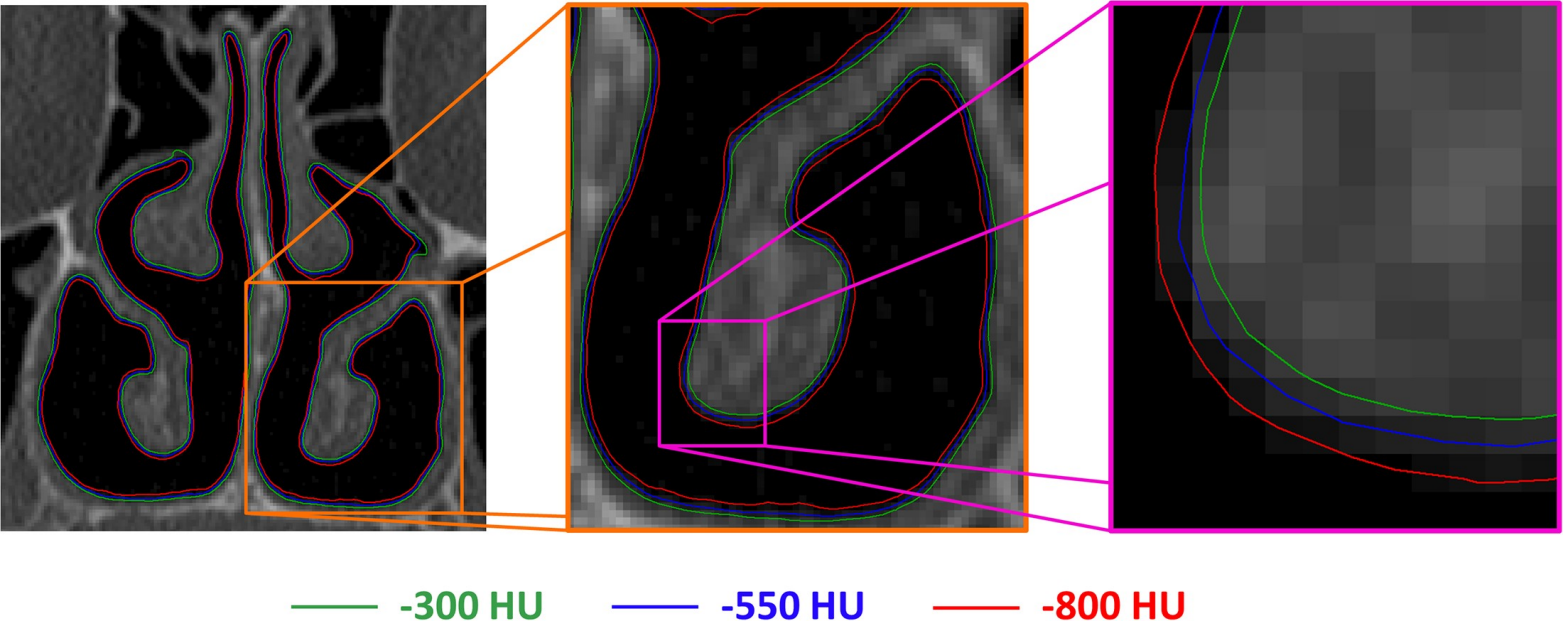
Outline of 3D reconstructions obtained with three different thresholds. Coronal CT scan of patient #1 showing the outlines of the 3D reconstructions created with segmentation thresholds -300 HU (green), -550 HU (blue), and -800 HU (red). The close-up view (right-side panel) reveals a nearly uniform distance of 1 to 2 pixels between the models created with segmentation thresholds -300 HU and -800 HU.

**Fig 4:**
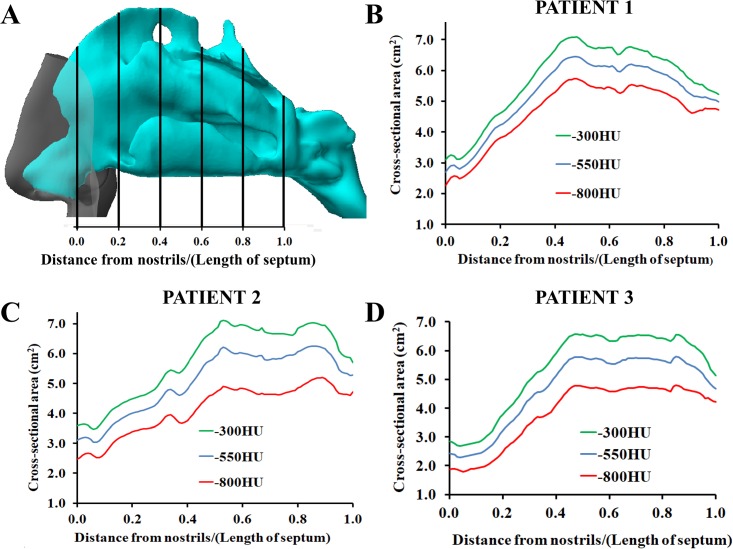
Airspace cross-sectional areas after mucosal decongestion with Oxmetazoline as a function of distance from nostrils. (A) Definition of the relative distance from nostrils. (B,C,D) In all three patients, the airspace cross-sectional area increased systematically throughout the nasal cavity as the segmentation threshold was increased from -800HU to -300HU.

The thresholding range had a substantial impact on the volumes and cross-sectional areas of the 3D models. In the turbinate region (coronal section at relative distance D = 0.5), the distance between the contours of the 3D models created with thresholds of -800HU and -300HU was approximately 0.4 to 0.6 mm, which corresponds to a gap of 1 to 2 pixels surrounding the entire perimeter (Fig 3). The volume of the nasal airspace (unilateral, nostrils to choana) increased from an average of 12.6 ± 1.1ml to 16.9 ± 1.3ml (a 34% increase) when the segmentation threshold was increased from -800HU to -300HU (Table 2). The surface area (unilateral, nostrils to choana) increased from an average of 83.1 ± 5.5 cm^2^ to 87.6 ± 5.8cm^2^ (a 5% increase) for the same change in the segmentation threshold (Table 3). Plots of the airspace cross-sectional area (CSA) vs. distance from nostrils revealed that the average bilateral CSA in the turbinate region (0.2 ≤ D ≤ 1.0) increased from 4.61 ± 0.65 cm^2^ to 6.13 ± 0.77 cm^2^ (a 33% increase) when the threshold was increased from -800HU to -300HU (Fig 4).

**Table 2:** Unilateral volume of the human nasal cavity (nostrils to choana) in three patients with nasal airway obstruction (NAO) after mucosal decongestion with Oxmetazoline.

Unilateral Volume (ml)				
Patient	Cavity	-800HU	-550HU	-300HU
**1**	Left	12.6	14.2 (12.5%)	15.6 (23.6%)
**2**		12.4	15.4 (24.2%)	17.6 (41.6%)
**3**		11.0	13.5 (22.7%)	15.5 (41.0%)
**1**	Right	13.8	15.4 (11.7%)	16.8 (22.0%)
**2**		13.8	16.8 (21.3%)	19.0 (37.4%)
**3**		11.9	14.6 (22.2%)	16.7 (40.0%)
**Average volume (ml)**		12.6 ± 1.1	15.0 ± 1.1	16.9 ± 1.3
**Average volume increase (%)**			19.1% ± 5.5%	34.3% ± 9.0%

Nasal cavity volume is strongly dependent on the threshold used for segmentation of the airspace from CT scans (-800 HU, -550 HU, or -300 HU). Numbers in parenthesis represent the percentage increase compared to models created with a threshold of -800HU.

**Table 3:** Unilateral surface area (cm^2^) of the human nasal cavity (nostrils to choana) in three patients with nasal airway obstruction (NAO) after mucosal decongestion with Oxmetazoline.

Unilateral Surface Area (cm^2^)				
Patient	Cavity	-800HU	-550HU	-300HU
**1**	Left	79.6	80.8 (1.5%)	81.8 (2.8%)
**2**		89.8	94.0 (4.7%)	94.6 (5.3%)
**3**		78.1	81.5 (4.3%)	83.3 (6.7%)
**1**	Right	81.7	82.9 (1.4%)	84.1 (3.0%)
**2**		90.4	94.0 (4.0%)	94.9 (5.0%)
**3**		79.2	84.7 (7.0%)	86.5 (9.3%)
**Average area (cm^2^)**		83.1 ± 5.5	86.3 ± 6.1	87.6 ± 5.8
**Average area increase (%)**			3.8% ± 2.1%	5.3% ± 2.5%

A small, but consistent increase in surface area was observed as the threshold used for segmentation of the CT scans was increased from -800 HU to -300 HU. Numbers in parenthesis represent the percentage increase compared to models created with a threshold of -800HU.

### Effect of segmentation threshold on airflow variables

**Fig 5:**
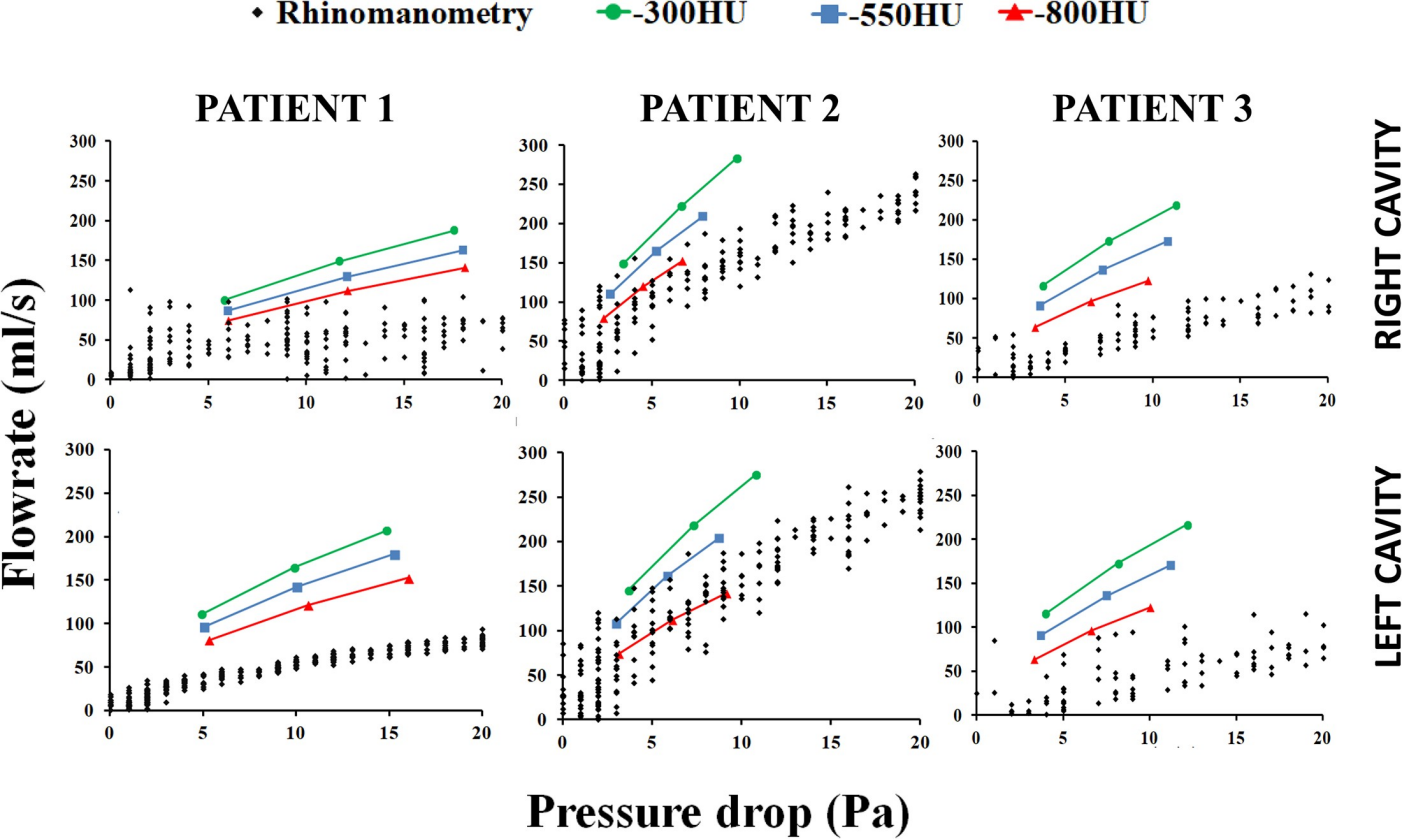
Flow-pressure curve measured with rhinomanometry and calculated with CFD in 3D models reconstructed with segmentation thresholds of -300HU, -550HU, and -800HU. Note the systematic increase in nasal airflow (reduction in nasal resistance) as the segmentation threshold increases from -800HU to -300HU.

These geometric differences between models created with different segmentation thresholds led to systematic differences in the flow-pressure curve obtained with CFD. Increasing the segmentation threshold from -800HU to -300HU led to a systematic increase in the flowrate in all models studied (Fig 5). The best agreement between CFD simulations and *in vivo* measurements (rhinomanometry) was obtained for the models created with segmentation threshold of -800HU (Fig 5). Unilateral nasal resistance at a unilateral flowrate of 125ml/s decreased from 0.078 ± 0.027Pa.s/ml to 0.038 ± 0.018Pa.s/ml when the segmentation threshold was increased from -800HU to -300HU (Table 4). On average, unilateral nasal resistance was approximately 51.9% ± 7.7% lower in the models created with segmentation threshold of -300HU as compared to models created with segmentation threshold of -800HU.

**Table 4:** Unilateral resistance (Pa.s/ml) of the human nasal cavity (nostrils to choana) measured at a unilateral flowrate of 125 ml/s in three patients with nasal airway obstruction (NAO) after mucosal decongestion with Oxmetazoline.

Unilateral Nasal Resistance (Pa.s/ml)				
Patient	Cavity	-800HU	-550HU	-300HU
**1**	Left	0.090	0.064 (-28.8%)	0.049 (-45.6%)
**2**		0.059	0.031 (-48.1%)	0.023 (-61.6%)
**3**		0.083	0.052 (-37.3%)	0.037 (-55.5%)
**1**	Right	0.118	0.091 (-23.0%)	0.069 (-41.8%)
**2**		0.039	0.026 (-32.3%)	0.020 (-48.9%)
**3**		0.080	0.049 (-38.9%)	0.034 (-57.9%)
**Average resistance (Pa.s/ml)**		0.078 ± 0.027	0.052 ± 0.024	0.038 ± 0.018
**Average resistance decrease (%)**			34.7% ± 8.7%	51.9% ± 7.7%

Unilateral nasal resistance was strongly dependent on the upper limit of the thresholding range used for segmentation of the CT scans (-800 HU, -550 HU, or -300 HU). Numbers in parenthesis represent the percentage reduction compared to models created with a threshold of -800HU.

**Fig 6:**
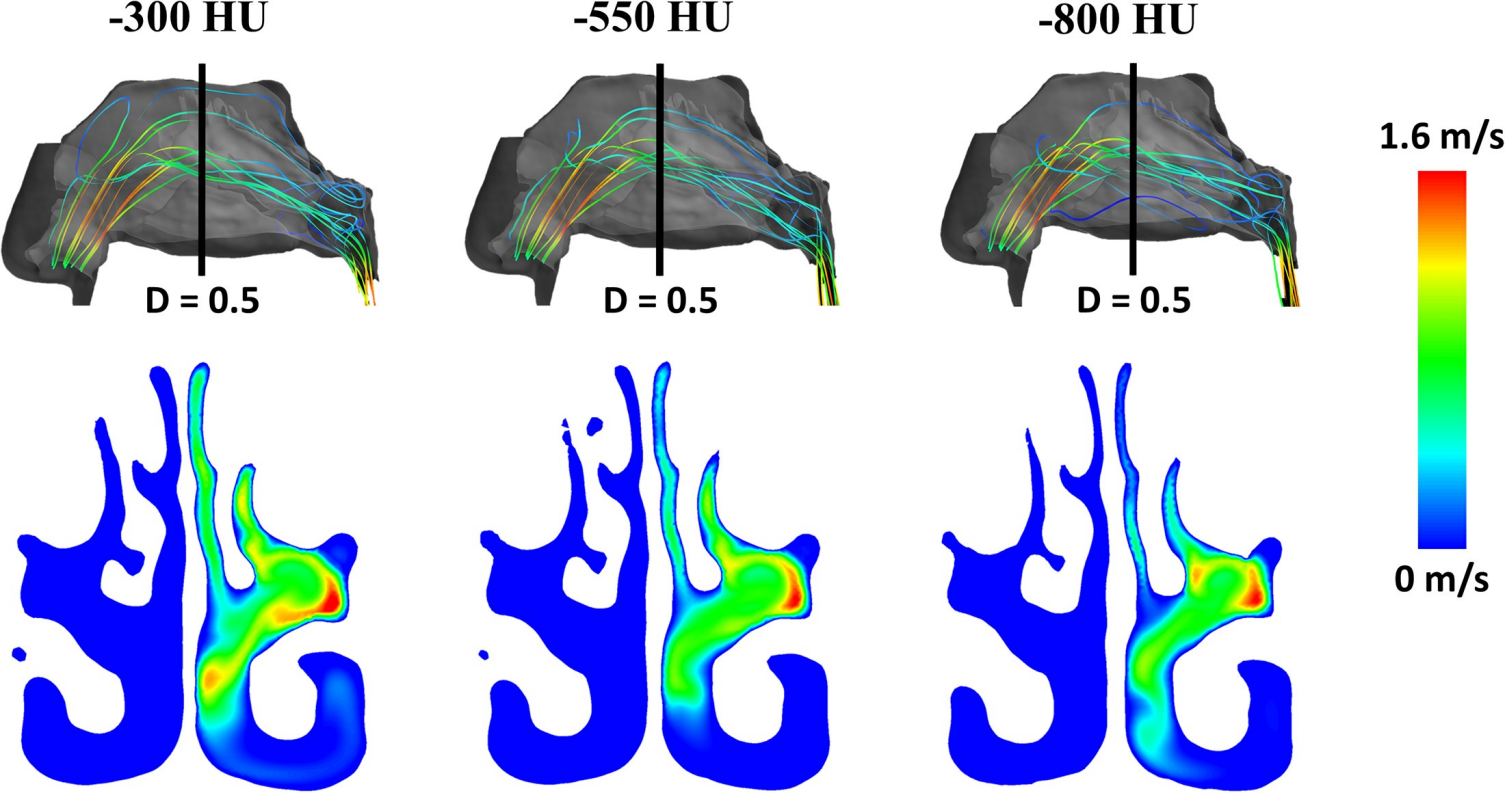
Inspiratory streamlines (top) and air velocity colormap at coronal section D = 0.5 (bottom). The main air stream flowed near the middle turbinate in patient #1 independently of the segmentation threshold (-300HU, -550HU, and -800HU). The right nostril was assumed to be blocked to reproduce rhinomanometry measurements of unilateral resistance in the left cavity (see text for details).

**Fig 7:**
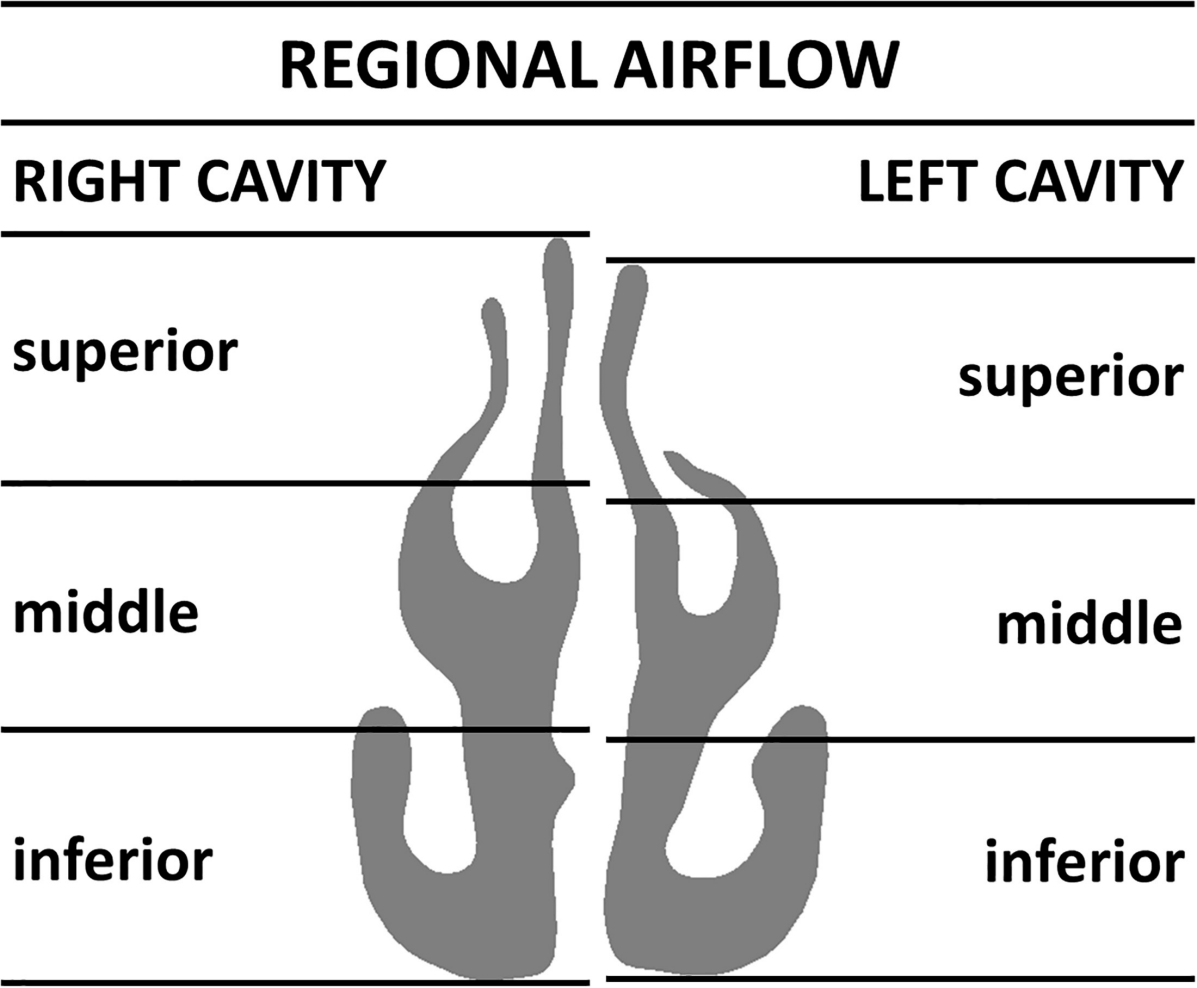
The coronal section D = 0.5 was divided in three regions (inferior, middle, superior) for the analysis of intranasal airflow distribution. The left and right cavities were analyzed independently. Each region corresponded to 1/3 of the nasal height. The inferior region corresponds to the nasal floor and the lower portion of the inferior turbinate. The middle region corresponds to the area surrounding the lower portion of the middle turbinate. The superior region corresponds to the olfactory cleft and upper portion of the middle meatus.

**Fig 8:**
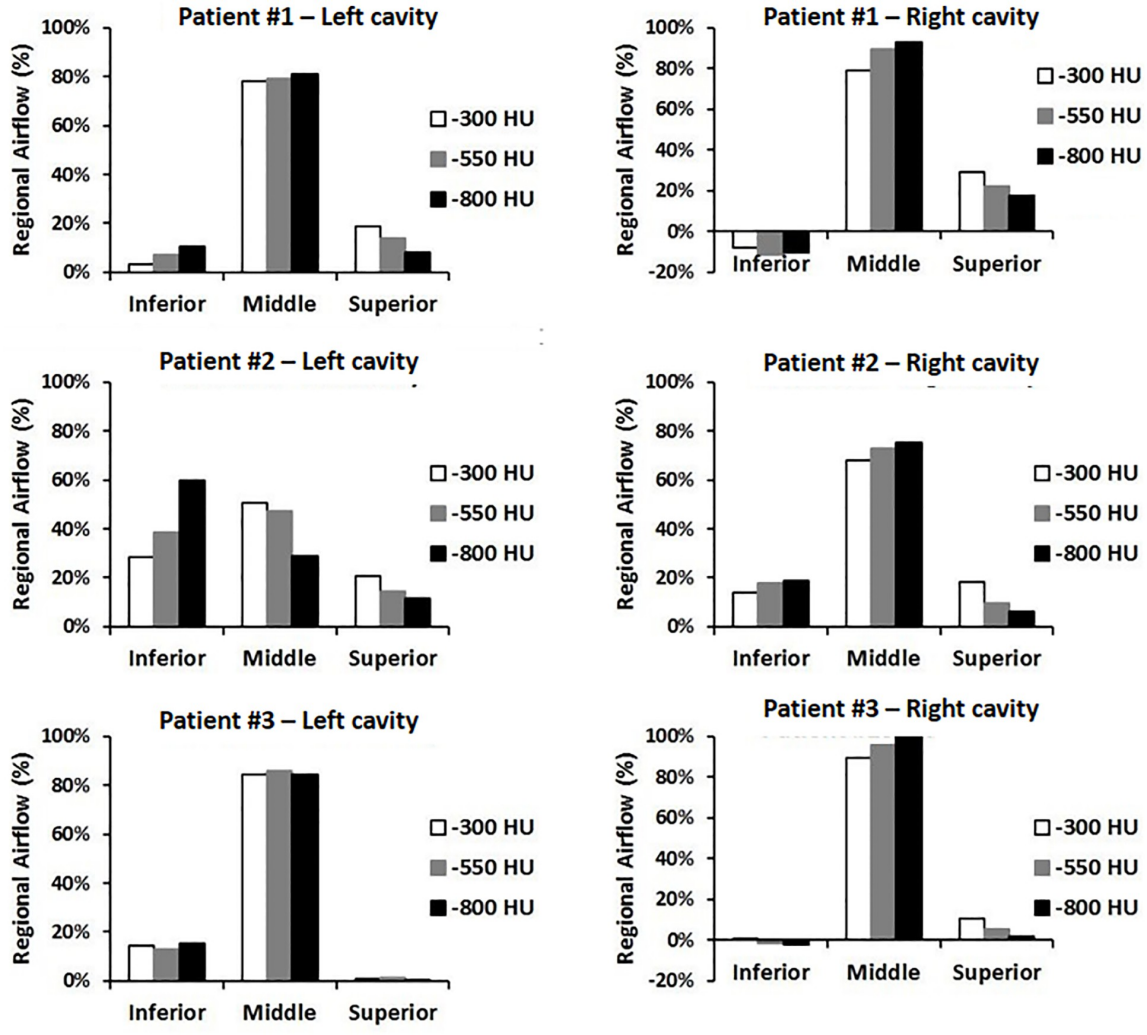
Intranasal airflow distribution at coronal section D = 0.5. In most nasal cavities, the middle region was the main airflow pathway. Intranasal airflow distribution was nearly independent of the segmentation threshold. Negative values correspond to regions of retrograde flow.

Finally, we studied the effect of the segmentation threshold on the intranasal airflow distribution (Figs 6, 7 and 8). Streamlines released from the exact same points at the nostril surface had essentially the same trajectories in the anterior third of the nose regardless of the segmentation threshold (Fig 6). Beyond the nasal valve, the streamlines followed different trajectories in models created with different segmentation thresholds, but intranasal airflow distribution was not significantly affected by the segmentation threshold (Fig 6). The main flow path in these decongested noses was the region surrounding the middle turbinate (Fig 6). When the coronal section D = 0.5 was divided into three regions (inferior, middle, and superior) each corresponding to 1/3 of the nasal height (Fig 7), unilateral airflow allocation was on average (10.5 ± 17.1)% through the inferior region, (78.4 ± 17.2)% through the middle region, and (11.1 ± 7.4)% through the superior region in models created with a segmentation threshold of -550 HU. The segmentation threshold had a relatively minor impact on the intranasal airflow allocation (Fig 8).

## Discussion

Computer simulations of nasal airflow have many applications, such as quantifying the regional doses of nasal sprays [26–29] and virtual surgery planning for patients with nasal airway obstruction [6, 7, 9–12, 30, 31]. Many studies have evaluated how CFD-derived airflow variables are affected by numerical methods, including inlet boundary conditions [32], outlet boundary conditions [33, 34], flow regime (laminar or turbulent) [24], and assumption of transient or steady-state flow [35]. One underlying assumption of CFD models that is rarely considered in the literature is the accuracy of the anatomic model [13]. In the early years of CFD simulations of nasal airflow, idealized anatomic models were sometimes used [36, 37]. As the field progresses towards patient-specific models for surgical planning [12], it is necessary to quantify the sensitivity of CFD variables to uncertainty in the 3D reconstruction of the nasal anatomy, which can be caused by changes in nasal mucosa engorgement due to the nasal cycle [38, 39], but can also be due to uncertainty in the airway segmentation from medical images.

The nasal cavity is the most challenging part of upper airway to be segmented [40]. Its manual segmentation is time consuming [41] and suffers from inter-individual and intra-individual variability [42]. Most CFD publications in the literature have used a semi-automated approach to segment the nasal airways. Automatic methods for segmenting the nasal airspace were recently described [30, 43, 44]. These automatic methods can generate 3D reconstructions that are equivalent to manual segmentation performed by a clinician [44], but it is unclear whether manual segmentation should be used as a ground truth. The gold standard should be a comparison between CFD simulations and *in vivo* measurements of nasal airflow performed on the same patient. However, few studies to date have compared patient-specific CFD simulations and *in vivo* measurements of nasal airflow.

Manual segmentation of the nasal airspace starts with selecting a CT number threshold to identify the air-tissue boundary. However, an optimal threshold for good correlation between CFD simulations and *in vivo* measurements of nasal airflow is lacking. Kawari and coauthors [19] recommended a threshold of -30HU to segment the nasal airway based on the volume of silicone required to fill the maxillary sinus of a *Macaca fuscata* specimen. However, when applied to humans this segmentation threshold predicted volumes of the paranasal sinuses that were too large. Nardelli and colleagues [45] reported that an optimal threshold to segment the tracheal airway is -800HU. Zeiberg and coauthors [20], based on air segmentation of a plastic conic phantom, concluded that an optimal threshold for air segmentation is between -500HU and -300HU. This is similar to the threshold of -460HU suggested by Nakano and colleagues [18] based on a phantom made of acrylic resin and urethane resin. These studies illustrate the lack of consensus for the optimal threshold for segmenting the nasal airspace from CT scans.

In our study, the segmentation threshold had a minor impact on the shape of the nasal cavity and on its surface area (Table 3). However, airspace cross-sectional areas (Fig 4) and volume (Table 2) were highly sensitive to the segmentation threshold. This variation in nasal cavity size had a significant impact on resistance, which decreased 52% when the segmentation threshold increased from -800HU to -300HU (Table 4, Fig 5). These results are similar to those reported by Quadrio and coauthors [13], who tested a smaller range of segmentation thresholds (namely -280HU to -120HU), but also observed a systematic reduction in nasal resistance when the segmentation threshold was increased. Therefore, quantitative agreement between patient-specific CFD simulations and *in vivo* measurements of nasal airflow will likely require the identification of an optimal threshold for segmentation of the nasal airspace.

Some limitations of this study must be acknowledged. First, the sample size investigated was small (only 3 patients). Second, our study was not designed to determine the optimal threshold for segmentation of the nasal airway. Although our results suggest that a segmentation threshold of -800 HU provides the best correlation between CFD and rhinomanometry (Fig 5), determining the optimal segmentation threshold will require an appropriate study design. For example, the shape and volume of 3D reconstructions from CT scans can be compared to anatomical measurements performed *in vivo*, in cadavers, or in animal models [46]. Third, our study did not investigate the effect of the segmentation threshold on heat transfer, moisture transport, and particle transport in the nasal cavity. However, given the direct link between mass transport, flowrate, and transnasal pressure drop, it is likely that other CFD variables that are relevant to nasal physiology will also be sensitive to the segmentation threshold.

## Conclusion

In conclusion, our study illustrates that some anatomic variables (airspace cross-sectional area and volume) and CFD variables (pressure drop, flowrate, airflow resistance) are strongly dependent on the segmentation threshold, while other variables (surface area, intranasal flow distribution) are less sensitive to the segmentation threshold. Future research is needed to determine which radiodensity threshold provides optimal agreement between patient-specific CFD simulations and *in vivo* measurements performed on the same patient. We recommend that future CFD studies should always report the segmentation threshold used to reconstruct the nasal anatomy, since this will facilitate comparison of results between research groups and facilitate correlation between CFD simulations and *in vivo* measurements.
